# Pilot Implementation of a National, Web-Based Abortion Curriculum for Obstetrics–Gynecology Residents

**DOI:** 10.1097/og9.0000000000000165

**Published:** 2026-04-02

**Authors:** Biftu Mengesha, Elizabeth Lutz, AnnaMarie Connolly, Aliza Adler, Amanda Teal, Jennifer Keller, Erika Banks, Jody Steinauer

**Affiliations:** Department of Obstetrics, Gynecology, & Reproductive Sciences, University of California San Francisco, San Francisco, California; Department of Obstetrics and Gynecology, University of Mississippi Medical Center, Jackson, Mississippi; American College of Obstetricians & Gynecologists, Washington, DC; Department of Obstetrics and Gynecology, George Washington University School of Medicine and Health Sciences, Washington DC; and NYU Grossman Long Island School of Medicine, NYU Langone Hospital Long Island, Mineola, New York.

## Abstract

A pilot online, video-based abortion care curriculum nationally positively affected obstetrics–gynecology residents' clinical knowledge, providing residency programs with a useful, centralized educational resource.

Nationwide state-level abortion restrictions have significantly affected obstetrics–gynecology resident training. Although the Accreditation Council for Graduate Medical Education (ACGME) maintains that obstetrics–gynecology residents must be trained to provide comprehensive reproductive health care, including “access to clinical experience in the provision of abortions,”^1^ maintaining routine abortion training, both didactic and hands on, is challenging for programs in restricted states.^2,3^

After a 2022 national educational summit focused on abortion education and training with multiple educational and professional society stakeholders in obstetrics and gynecology, an abortion essentials curriculum, Patient-Centered Abortion Care Education, was developed by Innovating Education in Reproductive Education, the Ryan Residency Training Program in Abortion and Family Planning, and the American College of Obstetricians and Gynecologists. Patient-Centered Abortion Care Education is an online, self-directed curriculum hosted on the Innovating Education in Reproductive Education learning management system. Consisting of four modules designed for residents in both permissive and restrictive states, Patient-Centered Abortion Care Education contains 35 videos totaling approximately 7 hours and reviews patient-centered counseling, clinical knowledge, and technical skills essential to abortion care. This curriculum differs from other computer-based modules by its inclusion of realistic medical procedure animations and delivery by national clinical and educational experts in abortion care.

## METHODS

**Fig. 1.**
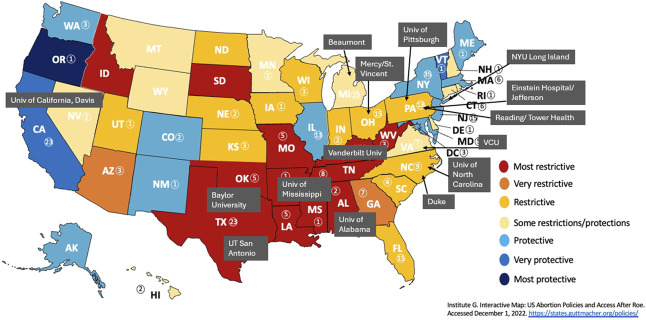
Obstetrics–gynecology residency programs included in the curriculum pilot implementation phase.

A convenience sample of 15 geographically diverse, ACGME-accredited obstetrics–gynecology residency programs was selected to participate in pilot implementation and evaluation of the curriculum (Fig. 1). Program directors were encouraged to offer the curriculum to all residents; however, their discretion was used on whether completion was mandatory. Between December 2022 and May 2023, participants completed a precurriculum survey in the learning management system, curricular modules, and a postcurriculum survey immediately after the didactic assessing perceived knowledge, satisfaction with and utility of the curriculum, and self-efficacy. The postcurriculum survey also included short qualitative questions assessing prior abortion education experiences, barriers experienced with abortion care restrictions, and specific feedback for future iterations. This pilot was deemed exempt from IRB review.

*Mengesha. Web-Based Abortion Curriculum for Obstetrics–Gynecology Residents. O&G Open 2026*.

## RESULTS

Of 118 residents completing the precurriculum survey, 67 (56.8%) completed all modules, and 55 (82.1%) of those participants completed the postcurriculum survey. Participants represented different training years, geographic regions, and programs from states with varying abortion restrictions (Table 1). Most participants either participated fully or planned on full participation in abortion care during training (n=85, 72.6%).

**Table 1.** Resident Participant Characteristics (N=118)

Demographic Characteristics	n (%)
Postgraduate year	
PGY1	36 (30.5)
PGY2	32 (27.1)
PGY3	29 (24.6)
PGY4	21 (17.8)
Location of training program based on abortion law permissiveness* (n=102)	
Most restrictive	30 (29.4)
Very restrictive	10 (9.8)
Restrictive	38 (37.2)
Very protective	13 (12.7)
Most protective	13 (12.7)
Plans for participation in abortion training during residency* (n=117)	
Participated fully/plans to participate fully	85 (72.6)
Partial participation/plans for partial participation	11 (9.4)
Opt out	6 (5.1)
Rotation canceled/not available	15 (12.8)
Plans to provide abortion care after residency for medical indications* (n=116)	
Certainly no	1 (0.1)
Probably no	7 (6.0)
Undecided	9 (7.7)
Probably yes	18 (15.5)
Certainly yes	81 (69.8)
Plans to provide abortion care after residency for any indication* (n=116)	
Certainly no	4 (3.4)
Probably no	11 (9.4)
Undecided	14 (12.1)
Probably yes	26 (22.4)
Certainly yes	61 (52.6)

* Categories with missing data.

Of the participants who completed the postcurriculum survey, the majority felt that the course met the stated learning objectives (n=51, 92.7%) and was extremely or somewhat useful (n=50, 90.9%). The clinical abortion care module had the highest utility rating, with 78.2% (n=43) rating it “extremely useful.” Most felt that the curriculum changed their knowledge regarding abortion (n=51, 92.7%). A significant minority (n=19, 34.5%) felt that the curriculum changed their beliefs about abortion, including their support for abortion, skills in abortion care, advocacy strengthening, or learning that it is a safer procedure than previously believed. However, the curriculum did not modify the majority of residents' intent to participate in abortion training. Findings were similar when stratified by training year. In short qualitative responses, participants appreciated curricular resources on medical and surgical abortion techniques, the patient counseling aspects, and the self-paced nature of the course. The most common critiques of the curriculum were the length of the course and the inclusion of content felt to be redundant such as videos on the importance and safety of abortion care.

## DISCUSSION

We piloted an online, video-based abortion care curriculum that positively affected residents' perceived knowledge. The Patient-Centered Abortion Care Education curriculum may provide obstetrics–gynecology residency programs with a useful, centralized educational resource for ACGME-required abortion care education, supplementing the loss of abortion training and education that programs experience in restricted states.

One strength of this pilot is its generalizability, with geographically diverse participating programs with varying abortion restrictions. Another strength includes ease of use for programs and trainees, which we hope will limit the administrative burden program that leadership and educators encounter when implementing new curricula. Limitations include the convenience sampling of programs and lower response rate of residents completing the full curriculum, which may have biased our results. In addition, residents with an interest in providing abortion care may have been more likely to participate fully in the curriculum.

Our pilot demonstrates that high-quality, accessible virtual curricula can meaningfully support resident education when clinical training opportunities are constrained. As training environments continue to shift, adaptable and learner-centered educational innovations like Patient-Centered Abortion Care Education may offer a practical strategy to maintain required competencies and to support trainee and future workforce education. Future directions include completion of a second, larger nationwide curriculum evaluation, which is currently underway, and curriculum validation, including modifying course content to meet the changing abortion landscape and trainee and program needs.
